# Cerebrovascular mortality: trend and seasonality in Brazilian capitals, 2000–2019

**DOI:** 10.11606/s1518-8787.2023057004813

**Published:** 2023-09-14

**Authors:** Luis Sauchay Romero, Ludmilla da Silva Viana Jacobson, Sandra de Souza Hacon

**Affiliations:** I Fundação Oswaldo Cruz Escola Nacional de Saúde Pública “Sergio Arouca” Rio de Janeiro RJ Brasil Fundação Oswaldo Cruz. Escola Nacional de Saúde Pública “Sergio Arouca”. Rio de Janeiro, RJ, Brasil; II Universidade Federal Fluminense Instituto de Matemática e Estatística Departamento de Estatística Niterói RJ Brasil Universidade Federal Fluminense. Instituto de Matemática e Estatística. Departamento de Estatística. Niterói, RJ, Brasil

**Keywords:** Mortality, Seasons, Cerebral Hemorrhage, Brain Ischemia

## Abstract

**OBJECTIVE:**

To evaluate the trend and seasonality of cerebrovascular mortality rates in the adult population of Brazilian capitals from 2000 to 2019.

**METHODS:**

This is an ecological and descriptive study of a time series of mortality due to cerebrovascular causes in adults (≥ 18 years) living in Brazilian capitals from 2000 to 2019, based on the Brazilian Mortality Information System. Descriptive statistical techniques were applied in the exploratory analysis of data and in the summary of specific, standardized rates and ratios by sociodemographic characteristics. The jointpoint regression model was used to estimate the trend of cerebrovascular mortality rates by gender, age groups, and geographic regions. The seasonal variability of rates by geographic regions was estimated using the generalized additive model by smoothing cubic splines.

**RESULTS:**

People aged over 60 years comprised 77% of all cerebrovascular deaths. Women (52%), white individuals (47%), single people (59%), and those with low schooling (57%, elementary school) predominated in our sample. Recife (20/1,000 inhab.) and Vitória (16/1,000 inhab.) showed the highest crude mortality rates. Recife (49/10,000 inhab.) and Palmas (47/10,000 inhab.) prevailed after we applied standardized rates. Cerebrovascular mortality rates in Brazil show a favorable declining trend for adults of all genders. Seasonality influenced rate increase from July to August in almost all region capitals, except in the North, which rose in March, April, and May.

**CONCLUSIONS:**

Deaths due to cerebrovascular causes prevailed in older single adults with low schooling. The trend showed a tendency to decline and winter, the greatest risk. Regional differences can support decision-makers in implementing public policies to reduce cerebrovascular mortality.

## INTRODUCTION

Cerebrovascular diseases (CBVD) feature among the first causes of death and permanent neurological sequelae in adults worldwide^1^. In 2019, about 6.55 million deaths occurred from CBVD globally (men: 3.33 million [95%CI: 3.04–3.62]; women: 3.22 million [95%CI: 2.86–3.54]), with little difference between ischemic (3.29 [95%CI: 2.93–3.61]) and hemorrhagic causes (3.27 million [95%CI: 2.91–3.61])^1^. In Latin America, CBVD also remained among the leading causes of death for decades, despite the steady decrease in the number of deaths from 1990 to 2019^1^.

Similarly, CBVD remain among the leading causes of death in Brazil, with a variable evolution in their trend over the last 30 years. Their spatial distribution also behaved unevenly in the country, with greater decreases in mortality rates in states with greater economic development. However, populations with low development indicators showed the greatest negative impacts^2^.

Epidemiological studies have contributed to the understanding and analysis of CBVD factors and determinants and to the support of policies, projects, and programs to promote health and prevent diseases. Thus, they better control this type of disease and reduce spending on specialized treatments and the psychological disorders of patients and families, especially in urban areas, which concentrate most of the population both worldwide and in Brazil (84% of the Brazilian population resides in urban areas and 24% in its capitals)^5^. In turn, metropolitan regions in Brazil have higher health coverage and better mortality records, enabling analyses in these areas to be representative of the population’s health condition^7,8^.

Studies on CBVD mortality often focus on trend analyses associated with epidemiological and sociodemographic profiles but few publications have considered their seasonal variability^2^. The impacts of temperature on cerebrovascular mortality have been widely documented. Epidemiological evidence suggests a strong U- or V-shaped relation between temperature and number of deaths, confirming the hypothesis that mortality progressively increases at extreme temperatures, both high and low^9^. The involved pathophysiological mechanisms, although still only partially understood, depend on individuals’ hydration state, sympathetic autonomic nervous system reactivity, activation of the renin-angiotensin-aldosterone system, and systemic inflammatory responses, which, together with other multiple risk factors (hypertension, obesity, diabetes mellitus, age, hypercholesterolemia, smoking, alcohol use, etc.), act negatively contributing to cause stroke^9^.

The analysis of the seasonal component, which directly impacts the exposed population, adds knowledge about the behavior and evolution of this disease in different periods of the year and configures a relevant indicator to predict and adopt control measures, especially in the current scenario of climate change, in which the great heterogeneity, complexity, and social, ecological, and climatic diversity of Brazilian municipalities and regions^10^ brings numerous challenges and impacts on the population’s health.

In this perspective, this study aimed to evaluate the trend and seasonality characteristics of CBVD mortality in adults aged over 18 years living in Brazilian capitals from 2000 to 2019 to update this epidemiological profile and provide objective and useful information to assist health decision-making.

## METHODS

This is an ecological and descriptive study of a time series of mortality due to cerebrovascular diseases occurring in the population (aged ≥ 18 years) living in the 26 Brazilian capitals and Federal District (DF) from 2000 to 2019. Capitals were grouped by geographic regions: South [SR]; Southeast [SER]; Northeast [NER]; North [NR]; and Midwest^2,6^. In 2010, the total population aged 18 years and above living in Brazilian capitals totaled 33,423,348 inhabitants^6^.

Daily deaths (codes I60 to I69 according to the 10th International Classification of Diseases) and the population estimates of each year (on July 1st), projected by the *Instituto Brasileiro de Geografia e Estatística* (IBGE), were obtained from the Datasus Information System of the Ministry of Health^6^. Descriptive statistical techniques were applied for data exploratory analysis and summary by age, gender (male and female), race (white; Black-Brown; Indigenous-yellow), marital status (single = single, separated or widowed; married = married or in a consensual union), and schooling (low = no schooling or complete elementary school; medium = complete high school; higher schooling = complete higher education). Health indicators, such as the sociodemographic characteristic and mortality rate ratios, were evaluated according to the equations below:


$$\text{ a) Ratio of deaths by gender }=\frac{\text{ male }}{\text{ female }}$$

$$\text{ b) Ratio of deaths by race }=\frac{\text{ white }}{\left(\text{ Black }+\text{ Brown })+(\text{ Indigenous }+\text{ yellow }\right)}$$

$$\text{ c) Ratio of deaths by marital status }=\frac{\text{ married }}{\text{ single }}$$

$$\text{ d) Ratio of deaths by schooling }=\frac{\text{ low schooling }}{\text{ medium }+\text{ higher schooling }}$$

$$\text{ e) Mortality rate }(\text{ period })=\frac{\text{ Total deaths by CBVD }}{\text{ Population in }2010+\text{ studied period }(20\text{ years })}\times 10,000\text{ inhab. }$$

$$\text{ f) Mortality rate }(\text{ annual })=\frac{\text{ Total annual deaths by CBVD }}{\text{ Estimated population }(\text{ July }1)}\times 10.000\text{ inhab. }$$

$$\text{ g) Mortality rate }(\text{ monthly })=\frac{\text{ Total monthly deaths by CBVD }}{\text{ Estimated population }(\text{ monthly })}\times 10,000\text{ inhab. }$$

To adjust the confounding effect in the comparisons of rates in the period between capital populations according to age groups (18–29 [young adults], 30–59 [adults], and ≥ 60 [older adults]), the direct method of standardization was used^11^. The WHO world population (2000–2025) was adopted as our standard population, thus defined to reflect the average age structure of the world population (https://seer.cancer.gov/stdpopulations/world.who.html).

To estimate the trend of annual cerebrovascular mortality rates adjusted by gender, age groups, geographic regions, and Brazil, the jointpoint regression mod*e*l was used^12^. This method selects the best fits from the sections of the continuous log-linear regression model and identifies the year(s) in which a trend change is produced, calculates the annual percent change (APC) between trend change points, and estimates the annual average percent change (AAPC) over the entire studied period. The number of junction points is obtained using the Monte Carlo permutation test (4,499 random permutations, significance level of 0.05). Once the k number of junction points is obtained, the different models are compared by the Bayesian Information Criterion.

Seasonality was also analyzed by geographic regions and the country. Descriptive statistical techniques were applied to observe the monthly and seasonal variability of the monthly time series of cerebrovascular mortality rate. Then, the mean values of the observations for each month of the year and their standard deviation with 95% confidence intervals were summarized.

For the temporal analysis, the generalized additive model (GAM)^13^ was used to estimate the seasonal variability of monthly cerebrovascular mortality rates over the studied period by smoothing cubic splines. GAM is used to interpret nonlinear relationships between variables based on nonparametric functions called smoothing curves, in which the form of association is defined by the data themselves. The basic structure fitted to model seasonal variability corresponds to the following equation:


g(μ)=α+s( month )+s( time )

in which g = additive binding function of the predictor variables; μ = mean value of monthly cerebrovascular mortality rates; α = intercept; s = smoothing spline function; month = month of the year; and time = number of months over the studied period. The models were evaluated by the adjustment coefficient (R^2^), deviance explained (DE), cross generalized validation (GCV), and residue analysis^14^.

A 5% significance level was set for the statistical tests. Microsoft Excel (version 2108), R (version 4.0.5 [packages: mgcv, ggplot2]), and jointpoint (version 4.9.0.1) were used for data downloading, management, statistical analysis, and graphical representation.

## RESULTS

During the studied period, 593,173 adults died from CBVD in Brazilian capitals, with a 2,474 mean monthly mortality (1st Quartile [Q1] = 2,371; 3rd Quartile [Q3] = 2,566). Older adults comprised 77% of all deaths. São Paulo and Rio de Janeiro, the largest cities in Brazil, showed the highest values during the studied period, with 120,124 and 91,987 deaths, respectively.

Regarding the sociodemographic characteristics of the total number of studied deaths, 23% of individuals were aged under 60 years and 52% were women. Regarding race, white (47%) individuals showed a higher incidence rate than Black people (44%). Regarding marital status, 59% of individuals were single. Low schooling also prevailed, 19% of victims had no education and 57% only attended elementary school.

In Table 1, death ratios by gender show that, in general, the highest mortality occurred in men, who were also more prevalent in the Brazilian South, Southeast, and Northeast, unlike the North and Midwest, which showed a prevalence of women. We found a higher number of men aged from 30 to 59 years (death ratios by gender: 1.07) in all capitals, with a greater predominance of this pattern in the North and Southeast. Women predominated among older adults (death ratios by gender: 0.83), except in the North. Regarding race, white individuals predominated in Southern capitals, especially Florianópolis; whereas Northeastern and Northern capitals showed a predominance of Black individuals, especially in Teresina, Belém, and Salvador. Marital status behaved uniformly, with a predominance of single people. The highest indicators of low schooling prevailed in Teresina and Palmas, with a 9.22 and 7.24 death ratio per schooling, respectively.

**Table 1:** Sociodemographic and epidemiological characteristics of mortality from cerebrovascular diseases in patients aged 18 years and above. Brazilian Capitals, 2000–2019.

Capitals	Population 2010	CBVD mortality	Death ratio				MR of the period	Age-standardized MR
				
	(≥ 18 years old)	n (%)	By gender	By race	By schooling	By marital status	(× 10,000 inhabitants)	(× 10,000 inhabitants)
South								
Porto Alegre	1,084,136	28,808 (4.86)	0.79	4.29	2.17	1.63	13.29	29.02
Florianópolis	327,561	4,121 (0.69)	0.9	10.79	2.48	1.22	6.29	17.8
Curitiba	1,320,385	19,415 (3.27)	0.92	8.66	2.5	1.22	7.35	20.49
Southeast								
São Paulo	8,411,089	120,124 (20.25)	0.91	2.31	3.08	1.43	7.14	18.19
Rio de Janeiro	4,815,996	91,987 (15.51)	0.84	1.3	2.32	1.71	9.55	20.62
Belo Horizonte	1,818,852	31,474 (5.31)	0.86	0.78	3.7	1.52	8.65	21.06
Vitória	250,027	8,158 (1.38)	0.97	0.98	4.05	1.32	16.31	41.65
Northeast								
Salvador	1,993,228	29,170 (4.92)	0.79	0.33	2.55	1.52	7.32	22.92
Aracaju	414,052	11,394 (1.92)	0.94	0.42	5.19	1.51	13.76	44.05
Maceió	648,527	17,832 (3.01)	0.98	0.5	5.84	1.36	13.75	44.79
Recife	1,140,476	45,149 (7.61)	0.92	0.55	3.6	1.48	19.79	49.32
João Pessoa	526,510	12,836 (2.16)	0.93	0.65	4.71	1.13	12.19	35.4
Natal	585,879	11,411 (1.92)	0.98	0.94	5.16	1.22	9.74	28.11
Fortaleza	1,762,994	30,754 (5.18)	0.94	0.5	4.6	1.12	8.72	26.63
Teresina	579,481	14,511 (2.45)	1	0.2	9.22	0.96	12.52	42.19
São Luis	717,173	14,416 (2.43)	0.96	0.44	4.08	1.19	10.05	36.36
North								
Palmas	154,133	2,570 (0.43)	1.11	0.35	7.24	0.99	8.34	46.56
Belém	992,891	21,525 (3.63)	0.95	0.32	3.01	1.08	10.84	33.35
Macapá	247,698	2,643 (0.45)	1.22	0.35	5.18	1.68	5.34	24.53
Boa Vista	181,411	1,949 (0.33)	1.31	0.39	2.91	1.28	5.37	24.37
Manaus	1,185,261	13,041 (2.20)	1.04	0.43	3.19	1.38	5.5	23.12
Rio Branco	217,280	3,024 (0.51)	1.19	0.35	6.07	1.38	6.96	26.88
Porto Velho	289,577	4,150 (0.70)	1.19	0.54	6.12	0.76	7.17	30.32
Midwest								
Cuiabá	395,410	6,688 (1.13)	1.12	0.46	3.25	1.31	8.46	27.28
Campo Grande	566,483	10,324 (1.74)	1.06	1.04	3.96	1.51	9.11	26.06
Goiânia	966,773	17,910 (3.02)	1.04	1.25	3.77	1.2	9.26	28.63
Brasília	1,830,065	17,789 (3.00)	0.95	1.02	2.97	1.58	4.86	17.63
Brazil								
Brazil	33,423,348	593,173 (100)	0.91	1.05	3.17	1.41	8.87	24.23

CBVD: cerebrovascular disease; %: percentage of mortality; MR: mortality rate.

Regarding mortality rates in the period, 13 capitals showed values above the national average, with the highest values in Recife (20/10,000 inhab.) and Vitória (16/10,000 inhab.). After standardization by age, results showed 20 capitals with values above the Brazilian average (24/10,000 inhab.), especially Recife (49/10,000 inhab.), Palmas (47/10,000 inhab.), Maceió (45/10,000 inhab.), and Aracaju (44/10,000 inhab.) (Table 1).

**Figure 1:**
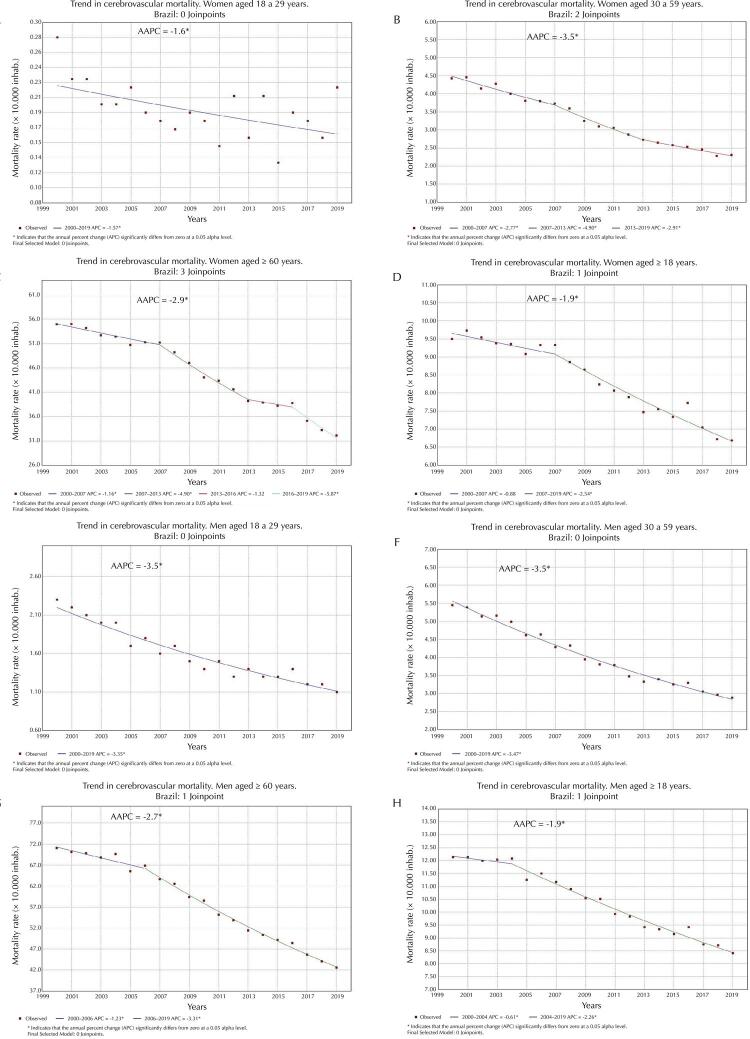
Trends in annual cerebrovascular mortality rates (per 10,000 inhabitants) in men and women according to age group. Brazil, 2000–2019.

On the other hand, Figure 1 shows that annual cerebrovascular mortality rates in Brazilian capitals had a favorable trend to decline for all genders and age groups, despite patterns variability across the regions in the country (Table 2). The APC and AAPC results in this table suggest that the variation in the changes of rate trend values relates more to differences between age groups, after comparison between genders, also showing variations, although less pronounced.

AAPC: average annual percentage change.

Note: A: women aged 18 to 29 years; B: women aged 30 to 59 years; C: women aged 60 years; D: women aged 18 years; E: men aged from 18 to 29 years; F: men aged from 30 to 59 years; G: men aged 60 years; H: men aged 18 years.

**Table 2:** Trends of annual cerebrovascular mortality rates (per 10,000 inhabitants) in men and women, according to age group. Brazilian region capitals, 2000–2019.

Region (Capitals)	Age	Female				Male			
	
		Period	Year(s) of trend change	APC (95%CI)	AAPC (95%CI)	Period	Year(s) of trend change	APC (95%CI)	AAPC (95%CI)
South (Florianópolis; Porto Alegre; Curitiba)	18–29	2000–2019			–5.8 (-17.2 to 7.2)	2000–2019			-1.1 (-3.9 to 1.9)
		2000–2007	2007	-19.0* (-27.6 to -9.3)		2000–2019		-1.1 (-3.9 to 1.9)	
		2007–2010	2010	26.4 (-45.7 to 194.5)					
		2010–2019		-3.9 (-11.1 to 3.8)					
	30–59	2000–2019			-5.8* (-6.4 to -5.1)	2000–2019			-4.8* (-6.1 to -3.4)
		2000–2019		-5.8* (-6.4 to -5.1)		2000–2004	2004	3.4 (-1.5 to 8.6)	
						2004–2013	2013	-8.5* (-10.1 to -7.0)	
						2013–2019		-4.2* (-6.7 to -1.7)	
	≥ 60	2000–2019			-3.9* (-4.7 to -3.0)	2000–2019			-3.9* (-4.9 to -2.8)
		2000–2007	2007	-1.2 (-3.3 to 0.9)		2000–2004	2004	0.0 (-4.9 to 5.2)	
		2007–2019		-5.4* (-6.3 to -4.5)		2004–2019		-4.9* (-5.5 to -4.2)	
	≥ 18	2000–2019			-4.0* (-4.8 to -3.2)	2000–2019			-4.0* (-4.9 to -3.0)
		2000–2007	2007	-1.5 (-3.5 to 0.5)		2000–2004	2004	0.1 (-4.4 to 4.8)	
		2007–2019		-5.4* (-6.8 to -4.6)		2004–2019		-5.0* (-5.6 to -4.4)	
Southeast (São Paulo; Rio de Janeiro; Belo Horizonte; Vitória)	18–29	2000–2019			-1.8* (-3.0 to -0.5)	2000–2019			-2.5* (-4.0 to -1.0)
		2000–2019		-1.8* (-3.0 to -0.5)				-2.5* (-4.0 to -1.0)	
	30–59	2000–2019			-4.4* (-4.7 to -4.1)	2000–2019			-4.0* (-4.4 to -3.6)
		2000–2019		-4.4* (-4.7 to -4.1)		2000–2007	2007	-5.2* (-6.1 to -4.2)	
						2007–2019		-3.3* (-3.8 to -2.9)	
	≥ 60	2000–2019			-3.3* (-3.6 to -3.0)	2000–2019			-3.4* (-3.8 to -3.0)
		2000–2019		-3.3* (-3.6 to -3.0)		2000–2009	2009	-2.7* (-3.4 to -2.0)	
						2009–2017		-4.0* (-4.5 to -3.4)	
	≥ 18	2000–2019			-1.8* (-2.0 to -1.5)	2000–2019			-1.8* (-2.0 to -1.7)
		2000–2019		-1.8* (-2.0 to -1.5)				-1.8* (-2.0 to -1.7)	
Northeast (Salvador; João Pessoa; Maceió; Teresina; São Luis; Aracajú; Recife; Natal; Fortaleza)	18–29	2000–2019			-1.6* (-2.9 to -0.2)	2000–2019			-1.2* (-2.5 to -0.2)
		2000–2019		-1.6* (-2.9 to -0.2)				-1.2* (-2.5 a-0.2)	
	30–59	2000–2019			-2.7* (-3.3 to -2.0)	2000–2019			-2.2* (-2.5 to -1.8)
Northeast (Salvador; João Pessoa; Maceió; Teresina; São Luis; Aracajú; Recife; Natal; Fortaleza)		2000–2005	2005	-0.5 (-2.8 to -1.9)		2000–2019		-2.2* (-2.5 to -1.8)	
		2005–2019		-3.4* (-3.9 to -3.0)					
	≥ 60	2000–2019			-2.4* (-2.8 to -2.0)	2000–2019			-2.2* (-2.6 to -1.8)
		2000–2006	2006	-0.1 (-1.4 to 1.2)		2000–2005	2005	0.4 (-1.0 to -1.8)	
		2006–2019		-3.5* (-3.8 to -3.1)		2005–2019		-3.1* (-3.4 to -2.8)	
	≥ 18	2000–2019			-0.5* (-0.9 to -0.0)	2000–2019			-0.5* (-0.7 to -0.3)
		2000–2006	2006	1.0 (-0.3 to 2.3)		2000–2019		-0.5* (-0.7a -0.3)	
		2006–2019		-1.2* (-1.6 to -0.8)					
North (Palmas; Boa Vista; Belém; Porto Velho; Manaus; Macapá; Rio Branco)	18–29	2000–2019			1.1 (-1.3 to 3.5)	2000–2019			-0.4 (-3.0 to 2.3)
		2000–2019		1.1 (-1.3 to 3.5)		2000–2019		-0.4 (-3.0 to 2.3)	
	30–59	2000–2019			-2.2* (-2.7 to -1.6)	2000–2019			-2.3* (-2.8 to -1.9)
		2000–2019		-2.2* (-2.7 to -1.6)		2000–2019		-2.3* (-2.8 to -1.9)	
	≥ 60	2000–2019			-1.9* (-2.4 to -1.4)	2000–2019			-1.7* (-2.4 to -1.1)
		2000–2019		-1.9* (-2.4 to -1.4)		2000–2015	2015	-1.0* (-1.4 to -0.6)	
						2015–2019		-4.5* (-7.5 to -1.5)	
	≥ 18	2000–2019			-0.1 (-0.5 to 0.3)	2000–2019			0.1 (-0.2 to 0.4)
				-0.1 (-0.5 to 0.3)				0.1 (-0.2 to 0.4)	
Midwest (Campo Grande; Cuiabá; Goiânia; Brasília)	18–29	2000–2019			-1.4 (-3.8 to 1.2)	2000–2019			-1.2 (-3.4 to 1.0)
		2000–2019		-1.4 (-3.8 to 1.2)		2000–2019		-1.2 (-3.4a 1.0)	
	30–59	2000–2019			-3.9* (-4.4 to -3.3)	2000–2019			-3.7* (-4.1 to -3.2)
		2000–2019		-3.9* (-4.4 to -3.3)		2000–2019		-3.7* (-4.1 to -3.2)	
	≥ 60	2000–2019			-2.7* (-4.1 to -1.3)	2000–2019			-3.0* (-3.5 to -2.6)
		2000–2009	2009	-1.8* (-2.5 to -1.1)		2000–2019		-3.0* (-3.5 to -2.6)	
		2009–2013	2013	-5.7* (-9.4 to -1.9)					
		2013–2016	2016	1.0 (-6.6 to 9.3)					
		2016–2019	2019	-5.0* (-8.6 to -1.2)					
	≥ 18	2000–2019			-0.2* (-0.6 to -0.2)	2000–2019			-1.0* (-1.4 to -0.6)
		2000–2019		-0.2* (-0.6 to -0.2)		2000–2019		-1.0* (-1.4 to -0.6)	

APC: annual percent change; AAPC: average annual percentage change; 95%CI: 95% confidence interval.

* Significantly different from zero (p < 0.05)

Considering geographical distribution, the results in Table 2 show that the most significant reductions in the trend of average annual percentages over the studied period occurred in women aged 30 to 59 years in Southern (-5.8*[-6.4; -5.1]) and Southeastern (-4.4*[-4.7; -4.1]) capitals. In older adults (the group with the highest mortality rate among women, with rates above 31 deaths per 10,000 inhab.) (Figure 1C), the South (-4.0*[-.48; -3.2]) and Southeast (-3.3*[-3.6; -3.0]) also showed greater declines. The behavior among men was similar, with the largest significant reductions in AAPC at ages 30 to 59 years in Southern (-4.8*[-6.1; -3.4]) and Southeastern (-4.0*[-4.4; -3.6]) capitals, with rates above 42 deaths per 10,000 inhab. for older adults (Figure 1G). Northern and Northeastern capitals generally showed the smallest trend decreases.

**Figure 2:**
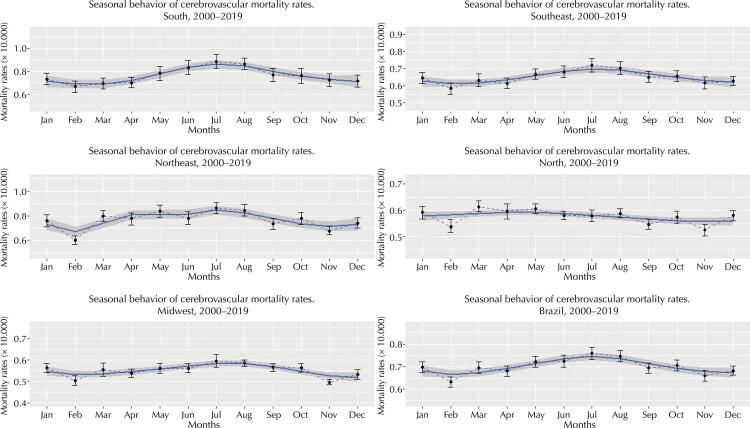
Cerebrovascular mortality rate seasonality. Brazil and regions, 2000–2019.

Note: the blue solid line shows the smoothing curves of the behavior of mortality rates, estimated by the generalized additive models (GAM); whereas gray areas, 95% confidence intervals. Dashed and dotted lines with an error bar show mean behavior with the 95% confidence intervals of cerebrovascular mortality rates.

Regarding seasonal behavior, we can establish that the smoothing curves estimated by GAM of cerebrovascular mortality rates as a function of the studied period fit seasonal variability, with a pattern consistent with the intervals of average behavior of these rates (Figure 2) in each region and Brazil. Table 3 shows the parameters after the application of generalized additive modeling. Note that the coefficients and effective degrees of freedom reflect the significance (p-value) if smoothing curve estimates, whose models showed reasonably adequate adjustments and prediction errors (GCV).

**Table 3:** Estimates of generalized additive model (GAM) parameters by Brazilian regions.

Macroregion	Explanatory variables	Coefficient	Effective degree of freedom	p-value	Model adjustment	GCV

					R^2^ / DE	
South	Intercept	0.7	-	< 0.001*	0.78 / 79.1%	0.0047
	f(month)	-	6.15	< 0.001*		
	f(time)	-	5.90	< 0.001*		
Southeast	Intercept	0.65	-	< 0.001*	0.83 / 84.3%	0.0013
	f(month)	-	7.60	< 0.001*		
	f(time)	-	6.77	< 0.001*		
Northeast	Intercept	0.88	-	< 0.001*	0.54 / 58.6%	0.0019
	f(month)	-	8.39	< 0.001*		
	f(time)	-	8.69	< 0.001*		
North	Intercept	0.58	-	< 0.001*	0.18 / 23.7%	0.0026
	f(month)	-	7.74	< 0.001*		
	f(time)	-	6.69	< 0.005*		
Midwest	Intercept	0.55	-	< 0.001*	0.42 / 46.3%	0.0021
	f(month)	-	8.23	< 0.001*		
	f(time)	-	7.47	< 0.001*		
Brazil	Intercept	0.70	-	< 0.001*	0.83 / 84.5%	0.0007
	f(month)	-	8.50	< 0.001*		
	f(time)	-	5.53	< 0.001*		

R^2^: coefficient of determination or adjustment of models; DE: deviation explained; GCV: generalized cross-validation; month: month of the year; time: number of months throughout the studied period.

* Significant.

In general, mortality rates showed their highest values in the middle of each year, pointing to July and August as offering the most risk (i.e., the winter period in the Southern Hemisphere), except for the Brazilian North, whose capitals showed the highest peaks from March to May. On the other hand, February showed the lowest rates in all Brazilian capitals and thus in the country, except for the North and Midwest.

We should mention that, except for the Brazilian North, the summer months in the Southern Hemisphere (December to February) showed the lowest values of mortality rates due to CBVD. However, the autumn (April to June) and spring (September to November) month behave as transition periods (Figure 2).

## DISCUSSION

The higher frequency of deaths in older adults confirms that age is an important variable, often associated with multiple risk factors: cerebral vascular atherosclerosis, arterial hypertension (AHT), diabetes mellitus and metabolic syndrome, smoking, among others, whose evolution, together with genetic and immunological factors, contribute to the deterioration of individuals’ health^1^. By cause of failures in adaptation responses due to internal changes in their regulatory systems, older adults are also more susceptible to environments with a high pollution load and extreme temperatures (minimum and maximum)^9^.

However, CBVD^9,15^also target young adults (with non-negligible mortality rates, as this study shows) despite a trend decline. Global incidence increases in those aged below 45 years have been reported, with the greatest impacts in low- and middle-income economies^15,16^. National studies have also characterized cerebrovascular morbidity and mortality in young people^14,17^, featuring among the foremost specific causes of death in those aged from 5 to 29 and 30 to 69 years^14^. Regarding risk factors, estimates suggest that the increased incidence of cerebrovascular episodes in young adults is related with the increasing trend of the aforementioned multiple risk factors. Other etiological factors that favor the occurrence of cerebrovascular episodes at these ages (also in Brazil) include HIV, sickle cell anemia, rheumatic heart disease, Chagas disease, tuberculosis, arterial dissection, and moyamoya disease^14^.

Several national studies have widely addressed the distribution of CBVD associated with gender, race, marital status, schooling, and years of potential life lost^4^. Lotufo et al.^18^, in a study on cerebrovascular mortality and race, found a predominance of Black And Brown individuals in Brazil, a result that contrasts with ours (in which white people showed the highest incidences), probably due to the age restriction (30–69 years) and short study period chosen by those authors. However, Dorlens et al.^2^reported the highest rates in older single men with low schooling from 1996 to 2015, results similar to ours.

In total, three characteristics resulting from our analyses enable us to consider the protective effect of the hormone estrogen, which eventually contributes to decreasing the risk of vascular accidents, increasing life expectancy in women. Firstly, the predominance of mortality rates in men aged 30 to 59 years, compared to the same group in women (Figures 1B and 1F); secondly, the larger significant reductions in the mortality trend in women aged 30 to 59 years compared to men of the same age group (Figures 1B and 1F); and finally, the increase in mortality rates in women aged above 60 years compared to those under it (Figures 1B and 1C). Estrogen has been reported as positively affecting the human immune system, stimulating the production of antibodies, regulating lymphopoiesis, increasing HDL cholesterol levels, lowering LDL, and relaxing, softening, and dilating blood vessels. It also shows antioxidant, anti-inflammatory, and cell membrane stabilizing properties^15,19^.

Our findings agree with the literature regarding mortality rate trends. Despite the increase in the absolute number of cases and incidence in some Brazilian states and municipalities, in general, a decline in mortality rates took place from the end of the 20th century to 2017, being more accentuated in regions with greater socioeconomic development and technological advances in high-complexity medicine^2^, a behavior also observed on a global scale^1,16^. In this line, Southern and Southeastern capitals, with the highest economic growth, outlined the largest reductions in APC and AAPC, unlike the less economically developed North and Northeast regions.

Taking Brazilian socioeconomic and health resource data in 2010 as reference, the Brazilian North and Northeast showed the lowest gross domestic product (NR GDP = R$ 12.7; NER GDP = R$ 9.6), per capita expenditures on public health actions and services in general (NR = R$ 572.88; NER = R$ 517.36), number of medical professionals (NR = 0.9/1,000 inhab.; NER = 1.02/1,000 inhab.), number of hospital beds (NR = 194/1,000 inhab.; NER = 2.29/1,000 inhab.), and number of neuroimaging devices for diagnosis: CT scanners (NR = 0.81 × 100,000 inhab.; NER = 0.88 × 100,000 inhab.) and magnetic resonance imaging (NR = 0.05/100,000 inhab.; NER = 0.02/100,000 inhab.)^20^.

On the other hand, the expansion of access to health services; the use of medications for acute cerebrovascular disease, chronic diseases, and lipid disorders; the greater control over modifiable risk factors; the increase in the number of intensive care units and availability of neuroimaging for diagnosis; and the implementation of promotion and prevention programs configure other factors that have favored the reduction of mortality rates^1,21,22^.

The National Health Survey (PNS)^23^ showed risk factors for CBVD above the national average for the Brazilian North and Northeast: the proportion of adults who never had their blood pressure measured (NR = 7%; NER = 4.2%), hospitalizations for AHT (NR = 14.6%; NER = 16%), hospitalizations for diabetes (NR = 14.2%; NER = 15.7%), and proportion of people with a previous diagnosis of CBVD (NR = 29.7%; NER = 26.4%). Other harmful risk factors with indicators below the national average include the proportion of hypertensive adults who had access to at least one drug in the Brazilian Popular Pharmacy Program (NR = 35%; NER = 28.7%), mean age of the beginning of alcohol consumption (NR = 18.5 years; NER = 18.3 years), and proportion of people with the recommended rates of physical activity (NR = 22.2%; NER = 22.3%).

Lee et al.^24^ found similar findings in South Korea (2011–2015), describing increased cerebrovascular mortality rates in regions with lower socioeconomic development and an association with overweight, alcohol consumption, number of hospital beds, and number of neuroimaging devices available to the population. Yanez et al.^25^also reported increases in poorer Colombian regions, with greater difficulties accessing pre-hospital and hospital health services and the correlation with AHT, obesity, and smoking. However, in the prefecture of Iwate (Japan), Omama et al.^22^ reported a decrease in the incidence rate trend of cerebrovascular diseases from 2008 to 2017 in those aged above and below 55 years of both genders.

Regarding seasonal behavior, the increase in rates in the middle of each year refers us, from the climatic point of view, to the fact that in the winter period, the risk of cerebrovascular mortality increases, specifically during July and August, except in Northern capitals. At this time of year, the country shows the lowest regional temperatures^26^. However, in Northern capitals (excluding their southern parts), the pattern of rate behavior differs, probably because the region shows no important minimum temperatures during this period, which are much higher (18° to 23°C) than in the rest of the country^26^.

During winter, we find multiple pathophysiological mechanisms linked to the occurrence of cardiovascular deaths: increased catecholamine levels, vasoconstriction, tachycardia, and blood pressure; hemoconcentration due to cold-related polyuria, and increased blood viscosity due to concentrations of coagulation factors, platelets, cholesterol, fibrinogen, and erythrocytes^9^.

Note that the risk of mortality persists in the summer, despite its lowest values. The increase in mortality in hot climate conditions is associated with extreme temperatures and heat waves, with more pronounced negative consequences in older adults^9^. Northern capitals behave unlike other Brazilian capitals as they follow the change in the climatic pattern, since September and October, the hottest months of the year in this region^27^, coincide with the lowest rates of cerebrovascular death, unlike other regions, in which rates decrease from December to February. However, the geographical location of capitals between the equatorial, tropical, and subtropical zones in Brazil suggests that the population, constantly exposed to environmental changes, adapts better to hot temperatures than to cold ones, regardless of other factors, which this this research ignored, that may determine this behavior.

In agreement with our results, both Keatinge et al.^27^ (in several European regions) and Su et al.^28^ (in 17 Chinese cities) reported a higher annual number of deaths and a relative risk of cold-related cardiovascular mortality, establishing regional differences and pointing to a small proportion of deaths unrelated to seasonal variability.

Silveira et al.^29^ concluded that both low and high temperatures in Brazil add to the risk of cardiovascular mortality in most Brazilian capitals, with variations according to geographic location. In general, the authors observed a U-shaped exposure-response relationship, with more pronounced consequences in low temperatures and places with greater thermal amplitude.

Regarding the potential limitations of this research, we should point out that the use of secondary data, subject to different levels of quality related to underreporting and ill-defined causes, may have partially influenced our results. Thus, regarding scope, coverage, and veracity, studies confirm important advances in the quality of mortality data in Brazil, especially in areas with greater socioeconomic development^2^. Data generated by the Mortality Information System (SIM) can reproduce the spatiotemporal dynamics of mortality, generating subsidies and priorities for policies and actions to promote health^14^. Another limitation to be considered is that the types of cerebral vascular arteries remained unspecified, despite the different pathophysiological mechanisms between them.

Our grouping of data by region configures another limitation, which probably reduces an accurate capture of local mortality behaviors. However, we find that this study estimates the significant trend and seasonality changes, enabling us to synthesize characteristics related to cerebrovascular mortality of interest to public health in areas with a high population concentration and subject to the influence of common environmental, social, and economic factors.

We conclude that our findings enabled us to characterize the epidemiological and sociodemographic profile of Brazilian capitals and the Federal District regarding cerebrovascular mortality from 2000 to 2019, pointing to single older adults of all with low schooling as the most affected population and, therefore, with a higher degree of social vulnerability. Depending on the region, white (South, Southeast) and Black individuals (North, Northeast, and Midwest) show the highest incidence rates. In the evaluation of the mortality rate trend, although it has decreased over the period, its behavior remains concerning due to the multifactorial character of its origin and evolution. Seasonality analysis showed winter as the period of highest risk of mortality in the national territory. Regional differences enable the definition of priorities and subsidizing decision-makers with medium and long-term actions toward planning and implementing policies and programs to reduce, promote, and prevent cerebrovascular mortality.

However, this study emphasizes the need to further develop and broaden research considering other risk factors outside the scope of this study.
