# Supplementary material for: Cerebrovascular mortality: trend and seasonality in Brazilian capitals, 2000–2019
Source: Rev Saude Publica. 2023 Sep 14;57:53. doi: 10.11606/s1518-8787.2023057004813 (PMC10519683; doi:10.11606/s1518-8787.2023057004813)
Supplement: Translation

# Translation: Mortalidade cerebrovascular: tendência e sazonalidade nas capitais brasileiras, 2000–2019

**Authors:** Luis Sauchay Romero, Ludmilla da Silva Viana Jacobson, Sandra de Souza Hacon

**Affiliations:** I Brasil Fundação Oswaldo Cruz. Escola Nacional de Saúde Pública “Sergio Arouca”. Rio de Janeiro, RJ, Brasil; II Brasil Universidade Federal Fluminense. Instituto de Matemática e Estatística. Departamento de Estatística. Niterói, RJ, Brasil

**Keywords:** Mortalidade, Estações do Ano, Hemorragia Cerebral, Isquemia Encefálica

## Abstract

**OBJETIVO:**

Avaliar a tendência e a sazonalidade das taxas de mortalidade cerebrovascular na população adulta das capitais brasileiras de 2000 a 2019.

**MÉTODOS:**

Estudo ecológico e descritivo de séries temporais de mortalidade por causas cerebrovasculares em adultos (≥ 18 anos) residentes nas capitais do Brasil no período 2000–2019, obtidas do Sistema de Informações sobre Mortalidade. Técnicas de estatística descritiva foram aplicadas na análise exploratória dos dados e no resumo de taxas específicas, padronizadas e razões por características sociodemográficas. A regressão de pontos de junção (*jointpoint regression model)* estimou a tendência das taxas de mortalidade cerebrovascular por sexo, grupos etários e regiões geográficas. A variabilidade sazonal por regiões geográficas das taxas foi estimada utilizando o modelo aditivo generalizado por meio de *splines* de suavização cúbica.

**RESULTADOS:**

As pessoas maiores de 60 anos representaram 77% dos óbitos cerebrovasculares. Predominaram o sexo feminino (52%), a raça branca (47%), os solteiros (59%) e a baixa escolaridade (57%, ensino fundamental). As capitais Recife (20/1.000 hab.) e Vitória (16/1.000 hab.) apresentaram as maiores taxas brutas de mortalidade. Aplicando as taxas padronizadas Recife (49/10.000 hab.) e Palmas (47/10.000 hab.) prevaleceram. As taxas de mortalidade cerebrovascular no Brasil apresentam uma tendência favorável ao declínio em ambos os sexos e em adultos. A sazonalidade mostrou influenciar na elevação das taxas entre os meses de julho a agosto em quase todas as capitais das regiões, exceto na Norte, que se elevaram nos meses de março, abril e maio.

**CONCLUSÕES:**

Os óbitos por causa cerebrovascular prevaleceram em pessoas idosas, solteiras e com baixa escolaridade. A tendência foi favorável ao declínio, sendo o inverno o período de maior risco. As diferenças regionais permitem subsidiar os tomadores de decisões em relação à implementação de políticas públicas para reduzir a mortalidade cerebrovascular.

## INTRODUÇÃO

As doenças cerebrovasculares (DCBV) estão entre as primeiras causas de morte e sequelas neurológicas permanentes no adulto no mundo^1^. Em 2019 ocorreram aproximadamente 6,55 milhões de óbitos por DCBV globalmente (homens: 3,33 milhões [IC95% 3,04–3,62]; mulheres: 3,22 milhões [IC95% 2,86–3,54]), com pouca diferença entre os de causa isquêmica (3,29 milhões [IC95% 2,93–3,61]) e hemorrágica (3,27 milhões [IC95% 2,91–3,61])^1^. Na América Latina as DCBV também se mantiveram entre as primeiras causas de morte por décadas, apesar do decréscimo estável no número de óbitos entre 1990–2019^1^.

Similarmente no Brasil ainda estão entre as principais causas de morte, com uma evolução variável na tendência ao longo dos últimos 30 anos. A distribuição espacial também se comportou de maneira desigual no país, com maiores quedas das taxas de mortalidade nos estados com maior desenvolvimento econômico, entretanto os maiores impactos negativos foram observados em populações com baixos indicadores de desenvolvimento^2^.

Estudos epidemiológicos têm contribuído na compreensão e análise dos fatores e determinantes das DCBV, assim como no subsídio de políticas, projetos e programas de promoção da saúde e prevenção de doenças. Com isso, proporcionam um melhor controle desse tipo de doença, além da redução dos gastos em tratamentos especializados e transtornos psicológicos de pacientes e familiares, particularmente em zonas urbanas, onde se concentra a maior parte da população, tanto mundial quanto nacional (84% da população brasileira reside em zonas urbanas e 24% nas capitais)^5^. Por sua vez, no Brasil, as regiões metropolitanas apresentam maior cobertura de saúde e melhor qualidade nos registros de mortalidade, permitindo que as análises nessas áreas sejam representativas da situação de saúde^7,8^.

Os estudos sobre mortalidade por DCBV frequentemente são focados em análises de tendência associados a perfis epidemiológicos e sociodemográficos, porém poucas publicações consideraram a variabilidade sazonal desse evento^2^. Os impactos da temperatura na mortalidade cerebrovascular têm sido amplamente documentados. Evidências epidemiológicas sugerem que existe forte relação em forma de U (ou de V) entre a temperatura e o número de óbitos, confirmando a hipótese de que a mortalidade aumenta progressivamente em temperaturas extremas, tanto altas quanto baixas^9^. Os mecanismos fisiopatológicos envolvidos, embora não totalmente esclarecidos, dependem do estado de hidratação, da reatividade do sistema nervoso autônomo simpático, da ativação do sistema renina-angiotensina-aldosterona e da resposta inflamatória sistêmica, que junto a outros múltiplos fatores de risco (hipertensão, obesidade, diabetes mellitus, idade, hipercolesterolemia, fumo, uso de álcool etc.) refletem negativamente^9^.

A análise da componente sazonal, que impacta diretamente a população exposta, adiciona conhecimento sobre o comportamento e evolução dessa doença nos diferentes períodos do ano, além de ser um indicador relevante na previsão e adoção de medidas de controle, sobretudo no atual cenário de mudança climática, em que a grande heterogeneidade, complexidade, diversidade social, ecológica e climática dos municípios e regiões do Brasil^10^ traz inúmeros desafios e impactos sobre a saúde da população.

Nessa perspectiva, objetivou-se avaliar as características de tendência e sazonalidade da mortalidade por DCBV em adultos maiores de 18 anos, residentes nas capitais brasileiras no período de 2000 a 2019, com o propósito de atualizar o perfil epidemiológico e fornecer informações objetivas e úteis para auxiliar a tomada de decisões em saúde.

## MÉTODOS

Trata-se de um estudo ecológico e descritivo de séries temporais de mortalidade por doenças cerebrovasculares ocorridas na população (≥ 18 anos) residente nas 26 capitais do Brasil e no Distrito Federal (DF), no período 2000–2019. As capitais foram agrupadas por regiões geográficas: região Sul [RS]; região Sudeste [RSD]; região Nordeste [RND]; região Norte [RN]; e região Centro-Oeste [RCO]^2,6^. Em 2010, o total da população a partir de 18 anos de idade nas capitais era de 33.423.348 habitantes^6^.

Os óbitos diários, dos códigos I60 a I69, segundo a décima Classificação Internacional de Doenças, e as estimativas populacionais de cada ano (em 1º de julho) projetadas pelo Instituto Brasileiro de Geografia e Estatística (IBGE), foram obtidos do Sistema de Informações Datasus, do Ministério da Saúde^6^. Técnicas de estatística descritiva foram aplicadas para a análise exploratória e o resumo dos dados por idade, sexo (masculino e feminino), raça (branca; preta-parda; indígena-amarela), estado civil (solteiro = solteiro ou separado ou viúvo; casado= casado ou união consensual) e escolaridade (baixa = sem instrução ou ensino fundamental; média = ensino médio; superior = ensino superior). Foram avaliados indicadores de saúde, tais como razões por características sociodemográficas e taxas de mortalidade, calculadas de acordo com as equações abaixo:


$$\text{ a) Razão de óbitos por sexo }=\frac{\text{ masculino }}{\text{ feminino }}$$

$$\text{ b) Razão de óbitos por raça }=\frac{\text{ Raça branca }}{\left(\text{ raça preta }+\text{ parda })+(\text{ indigena }+\text{ amarela }\right)}$$

$$\text{ c) Razão deóbitos por estado civil }=\frac{\text{ solteiros }}{\text{ casados }}$$

$$\text{ d) Razão de óbitos por escolaridade }=\frac{\text{ baixa escolaridade }}{\text{ escolaridade média }+\text{ superior }}$$

$$\text{ e) Taxa de mortalidade }(\text{ do perlodo })=\frac{\text{ Total de mortes por }DCBV}{\text{ População de }2010+\text{ perlodo de estudo }(20\text{ anos })}\times 10.000hab\text{. }$$

$$\text{ f) Taxa de mortalidade }(\text{ anual })=\frac{\text{ Total de mortes anuais por DCBV }}{\text{ População estimada }(1\text{ de julho })}\times 10.000\text{ hab. }$$

$$\text{ g) Taxa de mortalidade (mensal) }=\frac{\text{ Total de mortes mensais por DCBV }}{\text{ Populaçấo estimada }(\text{ mensal })}\times 10.000\text{ hab. }$$

Para ajustar o efeito do confundimento nas comparações das taxas do período entre as populações das capitais, segundo os grupos etários (18–29 [adulto jovem], 30–59 [adultos] e ≥ 60 [idosos]), se utilizou o método direto de padronização^11^. Adotou-se como população padrão a população mundial da OMS (2000–2025), definida para refletir a estrutura etária média da população mundial (https://seer.cancer.gov/stdpopulations/world.who.html).

Para estimar a tendência das taxas anuais de mortalidade cerebrovascular, ajustadas por sexo, grupos etários, regiões geográficas e para Brasil, se utilizou a regressão de pontos de junção (*jointpoint regression model*)^12^. O método seleciona os melhores ajustes do modelo de regressão log-linear contínuo por partes e identifica o(s) ano(s) em que uma mudança de tendência é produzida, calcula a variação percentual anual (*annual percent change* – APC) entre os pontos de mudança de tendência e estima a variação percentual média anual (*annual average percent change –* AAPC) em todo o período estudado. O número de pontos de junção é obtido usando o teste de permutação de Monte Carlo (4.499 permutações aleatórias, nível de significância de 0,05). Uma vez que o número k de pontos de junção são obtidos, os diferentes modelos são comparados pelo Critério de Informação Bayesiano (BIC).

A sazonalidade também foi analisada por regiões geográficas e para o País. Técnicas de estatística descritiva foram aplicadas para observar a variabilidade mensal e sazonal da série temporal taxa mensal de mortalidade cerebrovascular. Posteriormente foram resumidos os valores médios das observações para cada mês do ano e seu desvio padrão com 95% de intervalo de confiança.

Para a análise temporal utilizou-se o modelo aditivo generalizado (*generalized additive model –* GAM)^13^, o qual estimou a variabilidade sazonal das taxas mensais de mortalidade cerebrovascular ao longo do período de estudo por meio de *splines* de suavização cúbica. O GAM é usado na interpretação das relações não lineares entre variáveis, baseia-se em funções não paramétricas, denominadas curvas de alisamento, em que a forma de associação é definida pelos próprios dados. A estrutura básica ajustada para modelar a variabilidade sazonal corresponde à seguinte equação:


g(μ)=α+s( mês )+s( time )

Onde g = função de ligação aditiva das variáveis preditoras; µ = valor da média da taxa mensal de mortalidade cerebrovascular; α = intercepto; s = função *spline* de suavização cúbica; mês = mês do ano e time = número de meses ao longo do período do estudo. Os modelos foram avaliados pelo coeficiente de ajuste (R^2^), a deviação explicada (*deviance explained –* DE), a validação cruzada generalizada (*cross generalized validation –* CGV) e pelas análises de resíduos^14^.

Estabeleceu-se um nível de significância de 5% para os testes estatísticos. Os softwares Microsoft Excel (versão 2108), R (versão 4.0.5 [pacotes: mgcv, ggplot2]) e jointpoint (versão 4.9.0.1) foram utilizados para o *download*, manejo, análises estatísticas e representações gráficas dos dados.

## RESULTADOS

Durante o período do estudo 593.173 adultos foram a óbito por DCBV nas capitais do Brasil, com uma mortalidade média mensal de 2.474 (1ºQuartil [Q1] = 2.371; 3ºQuartil [Q3] = 2.566) casos. Os idosos representaram 77% dos óbitos. São Paulo e Rio de Janeiro, maiores cidades do País, atingiram os maiores valores durante o período do estudo, com 120.124 e 91.987, respectivamente.

Sobre as características sociodemográficas do total de óbitos estudados, 23% eram menores de 60 anos e 52% eram mulheres. Em relação à raça, a branca (47%) superou a preta (44%). Quanto ao estado civil, 59% eram solteiros. A baixa escolaridade também prevaleceu, sendo que 19% não tinham nenhum grau de instrução e 57% só tinham cursado o ensino fundamental.

Na Tabela 1, a razão de óbitos por sexo mostra que, em geral, a maior mortalidade ocorreu em homens, sendo eles também mais prevalentes nas regiões Sul, Sudeste e Nordeste, ao contrário da Norte e Centro-Oeste, onde prevaleceram as mulheres. Houve um maior número de homens entre 30 e 59 anos (razão de óbitos por sexo: 1,07) em todas as capitais, com maior predomínio desse padrão nas regiões Norte e Sudeste. Entre as pessoas idosas predominaram as mulheres (razão de óbitos por sexo: 0,83), exceto no Norte. Quanto à raça, a branca predominou nas capitais do Sul, com destaque para Florianópolis; nas regiões Nordeste e Norte houve predomínio da preta, se destacando as capitais Teresina, Belém e Salvador. O estado civil se comportou de maneira uniforme, com predomínio dos solteiros. Em Teresina e Palmas prevaleceram os maiores indicadores de baixa escolaridade, com razões de óbitos por escolaridade de 9,22 e 7,24 respectivamente.

**Tabela 1:** Características sociodemográficas e epidemiológicas da mortalidade por doenças cerebrovasculares em pacientes de 18 anos e mais. Capitais do Brasil, período 2000-2019.

Capitais	População 2010	Mortalidade DCBV	Razão de óbito				TM do período	TM padronizada por idade
				
	(≥ 18 anos)	n (%)	Por sexo	Por raça	Por escolaridade	Por estado civil	(× 10.000 hab.)	(× 10.000 hab.)
Região Sul								
Porto Alegre	1.084.136	28.808 (4,86)	0,79	4,29	2,17	1,63	13,29	29,02
Florianópolis	327.561	4.121 (0,69)	0,9	10,79	2,48	1,22	6,29	17,8
Curitiba	1.320.385	19.415 (3,27)	0,92	8,66	2,5	1,22	7,35	20,49
Região Sudeste								
São Paulo	8.411.089	120.124 (20,25)	0,91	2,31	3,08	1,43	7,14	18,19
Rio de Janeiro	4.815.996	91.987 (15,51)	0,84	1,3	2,32	1,71	9,55	20,62
Belo Horizonte	1.818.852	31.474 (5,31)	0,86	0,78	3,7	1,52	8,65	21,06
Vitória	250.027	8.158 (1,38)	0,97	0,98	4,05	1,32	16,31	41,65
Região Nordeste								
Salvador	1.993.228	29.170 (4,92)	0,79	0,33	2,55	1,52	7,32	22,92
Aracaju	414.052	11.394 (1,92)	0,94	0,42	5,19	1,51	13,76	44,05
Maceió	648.527	17.832 (3,01)	0,98	0,5	5,84	1,36	13,75	44,79
Recife	1.140.476	45.149 (7,61)	0,92	0,55	3,6	1,48	19,79	49,32
João Pessoa	526.510	12.836 (2,16)	0,93	0,65	4,71	1,13	12,19	35,4
Natal	585.879	11.411 (1,92)	0,98	0,94	5,16	1,22	9,74	28,11
Fortaleza	1.762.994	30.754 (5,18)	0,94	0,5	4,6	1,12	8,72	26,63
Teresina	579.481	14.511 (2,45)	1	0,2	9,22	0,96	12,52	42,19
São Luis	717.173	14.416 (2,43)	0,96	0,44	4,08	1,19	10,05	36,36
Região Norte								
Palmas	154.133	2.570 (0,43)	1,11	0,35	7,24	0,99	8,34	46,56
Belém	992.891	21.525 (3,63)	0,95	0,32	3,01	1,08	10,84	33,35
Macapá	247.698	2.643 (0,45)	1,22	0,35	5,18	1,68	5,34	24,53
Boa Vista	181.411	1.949 (0,33)	1,31	0,39	2,91	1,28	5,37	24,37
Manaus	1.185.261	13.041 (2,20)	1,04	0,43	3,19	1,38	5,5	23,12
Rio Branco	217.280	3.024 (0,51)	1,19	0,35	6,07	1,38	6,96	26,88
Porto Velho	289.577	4.150 (0,70)	1,19	0,54	6,12	0,76	7,17	30,32
Região Centro-Oeste								
Cuiabá	395.410	6.688 (1,13)	1,12	0,46	3,25	1,31	8,46	27,28
Campo Grande	566.483	10.324 (1,74)	1,06	1,04	3,96	1,51	9,11	26,06
Goiânia	966.773	17.910 (3,02)	1,04	1,25	3,77	1,2	9,26	28,63
Brasília	1.830.065	17.789 (3,00)	0,95	1,02	2,97	1,58	4,86	17,63
Brasil								
Brasil	33.423.348	593.173 (100)	0,91	1,05	3,17	1,41	8,87	24,23

DCBV: doença cerebrovascular; %: percentual de mortalidade; TM: taxa de mortalidade.

Sobre as taxas de mortalidade do período, 13 capitais apresentaram valores acima da média do país, com os maiores valores em Recife (20/10.000 hab.) e Vitória (16/10.000 hab.). Após padronização por idade, os resultados mostraram 20 capitais com valores superiores à média do Brasil (24/10.000 hab.), se destacando Recife (49/10.000 hab.), Palmas (47/10.000 hab.), Maceió (45/10.000 hab.) e Aracaju (44/10.000 hab.) (Tabela 1).

Por outro lado, a Figura 1 mostra que nas capitais do Brasil as taxas anuais de mortalidade cerebrovascular tiveram uma tendência favorável ao declínio, tanto em ambos os sexos quanto em todos os grupos etários, ainda que mostrando padrões de comportamento variáveis nas diferentes regiões do País, como se mostra na Tabela 2. Os resultados dos APC e AAPC observados nessa tabela, sugerem que a variação nas mudanças nos valores da tendência das taxas está mais relacionada às diferenças entre as faixas etárias quando comparado ao comportamento entre ambos os sexos, em que também houve variações dos valores, porém menos acentuadas.

**Figura 1:** Tendência das taxas de mortalidade cerebrovascular (por 10.000 hab.) anual em homens e mulheres, segundo faixa etária. Brasil, período 2000–2019.

APC: variação percentual anual (do inglês *annual percent change*); AAPC: variação percentual média anual (do inglês *average annual percent change*).

Nota: A: mulheres de 18 a 29 anos; B: mulheres de 30 a 59 anos; C: mulheres ≥ 60 anos; D: mulheres ≥ 18 anos; E: homens de18 a 29 anos; F: homens de 30 a 59 anos; G: homens ≥ 60 anos; H: homens ≥ 18 anos.

**Tabela 2:** Tendência das taxas anuais de mortalidade cerebrovascular (por 10.000 hab.) em homens e mulheres, segundo faixa etária. Capitais das regiões do Brasil, período 2000–2019.

Região (Capitais)	Idade	Feminino				Masculino			
	
		Período	Ano(s) de variação da tendência	APC (IC95%)	AAPC (IC95%)	Período	Ano(s) de variação da tendência	APC (IC95%)	AAPC (IC95%)
Região Sul (Florianópolis; Porto Alegre; Curitiba)	18–29	2000–2019			–5,8 (-17,2 a 7,2)	2000–2019			-1,1 (-3,9 a 1,9)
		2000–2007	2007	-19,0* (-27,6 a -9,3)		2000–2019		-1,1 (-3,9 a 1,9)	
		2007–2010	2010	26,4 (-45,7 a 194,5)					
		2010–2019		-3,9 (-11,1 a 3,8)					
	30–59	2000–2019			-5,8* (-6,4 a -5,1)	2000–2019			-4,8* (-6,1 a -3,4)
		2000–2019		-5,8* (-6,4 a -5,1)		2000–2004	2004	3,4 (-1,5 a 8,6)	
						2004–2013	2013	-8,5* (-10,1 a -7,0)	
						2013–2019		-4,2* (-6,7 a -1,7)	
	≥ 60	2000–2019			-3,9* (-4,7 a -3,0)	2000–2019			-3,9* (-4,9 a -2,8)
		2000–2007	2007	-1,2 (-3,3 a 0,9)		2000–2004	2004	0,0 (-4,9 a 5,2)	
		2007–2019		-5,4* (-6,3 a -4,5)		2004–2019		-4,9* (-5,5 a -4,2)	
	≥ 18	2000–2019			-4,0* (-4,8 a -3,2)	2000–2019			-4,0* (-4,9 a -3,0)
		2000–2007	2007	-1,5 (-3,5 a 0,5)		2000–2004	2004	0,1 (-4,4 a 4,8)	
		2007–2019		-5,4* (-6,8 a -4,6)		2004–2019		-5,0* (-5,6 a -4,4)	
Região Sudeste (São Paulo; Rio de Janeiro; Belo Horizonte; Vitória)	18–29	2000–2019			-1,8* (-3,0 a -0,5)	2000–2019			-2,5* (-4,0 a -1,0)
		2000–2019		-1,8* (-3,0 a -0,5)				-2,5* (-4,0 a -1,0)	
	30–59	2000–2019			-4,4* (-4,7 a -4,1)	2000–2019			-4,0* (-4,4 a -3,6)
		2000–2019		-4,4* (-4,7 a -4,1)		2000–2007	2007	-5,2* (-6,1 a -4,2)	
						2007–2019		-3,3* (-3,8 a -2,9)	
	≥ 60	2000–2019			-3,3* (-3,6 a -3,0)	2000–2019			-3,4* (-3,8 a -3,0)
		2000–2019		-3,3* (-3,6 a -3,0)		2000–2009	2009	-2,7* (-3,4 a -2,0)	
						2009–2017		-4,0* (-4,5 a -3,4)	
	≥ 18	2000–2019			-1,8* (-2,0 a -1,5)	2000–2019			-1,8* (-2,0 a -1,7)
		2000–2019		-1,8* (-2,0 a -1,5)				-1,8* (-2,0 a -1,7)	
Região Nordeste (Salvador; João Pessoa; Maceió; Teresina; São Luis; Aracajú; Recife; Natal; Fortaleza	18–29	2000–2019			-1,6* (-2,9 a -0,2)	2000–2019			-1,2* (-2,5 a -0,2)
		2000–2019		-1,6* (-2,9 a -0,2)				-1,2* (-2,5 a-0,2)	
	30–59	2000–2019			-2,7* (-3,3 a -2,0)	2000–2019			-2,2* (-2,5 a -1,8)
Região Nordeste (Salvador; João Pessoa; Maceió; Teresina; São Luis; Aracajú; Recife; Natal; Fortaleza		2000–2005	2005	-0,5 (-2,8 a -1,9)		2000–2019		-2,2* (-2,5 a -1,8)	
		2005–2019		-3,4* (-3,9 a -3,0)					
	≥ 60	2000–2019			-2,4* (-2,8 a -2,0)	2000–2019			-2,2* (-2,6 a -1,8)
		2000–2006	2006	-0,1 (-1,4 a 1,2)		2000–2005	2005	0,4 (-1,0 a -1,8)	
		2006–2019		-3,5* (-3,8 a -3,1)		2005–2019		-3,1* (-3,4 a -2,8)	
	≥ 18	2000–2019			-0,5* (-0,9 a -0,0)	2000–2019			-0,5* (-0,7 a -0,3)
		2000–2006	2006	1,0 (-0,3 a 2,3)		2000–2019		-0,5* (-0,7a -0,3)	
		2006–2019		-1,2* (-1,6 a -0,8)					
Região Norte (Palmas; Boa Vista; Belém; Porto Velho; Manaus; Macapá; Rio Branco)	18–29	2000–2019			1,1 (-1,3 a 3,5)	2000–2019			-0,4 (-3,0 a 2,3)
		2000–2019		1,1 (-1,3 a 3,5)		2000–2019		-0,4 (-3,0 a 2,3)	
	30–59	2000–2019			-2,2* (-2,7 a -1,6)	2000–2019			-2,3* (-2,8 a -1,9)
		2000–2019		-2,2* (-2,7 a -1,6)		2000–2019		-2,3* (-2,8 a -1,9)	
	≥ 60	2000–2019			-1,9* (-2,4 a -1,4)	2000–2019			-1,7* (-2,4 a -1,1)
		2000–2019		-1,9* (-2,4 a -1,4)		2000–2015	2015	-1,0* (-1,4 a -0,6)	
						2015–2019		-4,5* (-7,5 a -1,5)	
	≥ 18	2000–2019			-0,1 (-0,5 a 0,3)	2000–2019			0,1 (-0,2 a 0,4)
				-0,1 (-0,5 a 0,3)				0,1 (-0,2 a 0,4)	
Região Centro-Oeste (Campo Grande; Cuiabá; Goiânia; Brasília)	18–29	2000–2019			-1,4 (-3,8 a 1,2)	2000–2019			-1,2 (-3,4 a 1,0)
		2000–2019		-1,4 (-3,8 a 1,2)		2000–2019		-1,2 (-3,4a 1,0)	
	30–59	2000–2019			-3,9* (-4,4 a -3,3)	2000–2019			-3,7* (-4,1 a -3,2)
		2000–2019		-3,9* (-4,4 a -3,3)		2000–2019		-3,7* (-4,1 a -3,2)	
	≥ 60	2000–2019			-2,7* (-4,1 a -1,3)	2000–2019			-3,0* (-3,5 a -2,6)
		2000–2009	2009	-1,8* (-2,5 a -1,1)		2000–2019		-3,0* (-3,5 a -2,6)	
		2009–2013	2013	-5,7* (-9,4 a -1,9)					
		2013–2016	2016	1,0 (-6,6 a 9,3)					
		2016–2019	2019	-5,0* (-8,6 a -1,2)					
	≥ 18	2000–2019			-0,2* (-0,6 a -0,2)	2000–2019			-1,0* (-1,4 a -0,6)
		2000–2019		-0,2* (-0,6 a -0,2)		2000–2019		-1,0* (-1,4 a -0,6)	

APC: variação percentual anual (do inglês *annual percent change*); AAPC: variação percentual média anual (do inglês *average annual percent change*); IC95%: intervalo de confiança de 95%.

* Estatisticamente significativo (p < 0,05).

Considerando a distribuição geográfica, os resultados apresentados na Tabela 2 mostram que as reduções mais significativas da tendência em percentuais médios anuais ao longo do período do estudo foram em mulheres do grupo etário de 30 a 59 anos nas capitais das regiões Sul (-5.8*[-6.4; -5.1]) e Sudeste (-4.4*[-4.7; -4.1]). Nas idosas, grupo de maior representatividade na mortalidade entre as mulheres, com taxas acima de 31 óbitos por 10 mil habitantes (Figura 1C), as regiões Sul (-4.0*[-.48; -3.2]) e Sudeste (-3.3*[-3.6; -3.0]) também mostraram maiores declínios. Entre os homens o comportamento foi similar, com as maiores reduções significativas dos AAPC em idades de 30 a 59 anos nas capitais das regiões Sul (-4.8*[-6.1; -3.4]) e Sudeste (-4.0*[-4.4; -3.6]), com taxas superiores a 42 óbitos por 10 mil habitantes em idosos (Figura 1G). De maneira geral, os menores declínios da tendência foram exibidas nas capitais do Norte e Nordeste.

Com relação ao comportamento sazonal, pode-se afirmar que as curvas de alisamento estimadas pelo GAM da taxa de mortalidade cerebrovascular em função do período do estudo se ajustaram à variabilidade sazonal, cujo padrão condiz com os intervalos de comportamento médio dessas taxas (Figura 2) em cada região e para o Brasil. A Tabela 3 exibe os parâmetros resultantes da aplicação da modelagem aditiva generalizada. Nessa linha, deve-se destacar que os coeficientes e os graus de liberdade efetivos refletem a significância (p-valor) nas estimativas das curvas de alisamento, cujos ajustes e erros de predição (CGV) dos modelos foram razoavelmente adequados.

**Figura 2:** Sazonalidade da taxa de mortalidade cerebrovascular. Brasil e regiões, 2000 a 2019.

Nota: a linha sólida azul mostra as curvas de alisamento do comportamento das taxas de mortalidade estimadas pelos modelos aditivos generalizados (GAM) e as áreas cinzas o intervalo de confiança de 95%. As linhas tracejadas e pontilhadas com barra de erro mostram o comportamento médio e com intervalo de confiança de 95% da taxa de mortalidade cerebrovascular.

**Tabela 3:** Estimativas dos parâmetros dos modelos aditivos generalizados (GAM) por regiões do Brasil.

Região	Variáveis explicativas	Coeficiente	Grau de liberdade efetivo	p-valor	Ajuste do modelo	CGV

					R^2^ / DE	
Sul	Intercepto	0,7	-	< 0,001*	0,78 / 79,1%	0,0047
	f(mês)	-	6,15	< 0,001*		
	f(time)	-	5,90	< 0,001*		
Sudeste	Intercepto	0,65	-	< 0,001*	0,83 / 84,3%	0,0013
	f(mês)	-	7,60	< 0,001*		
	f(time)	-	6,77	< 0,001*		
Nordeste	Intercepto	0,88	-	< 0,001*	0,54 / 58,6%	0,0019
	f(mês)	-	8,39	< 0,001*		
	f(time)	-	8,69	< 0,001*		
Norte	Intercepto	0,58	-	< 0,001*	0,18 / 23,7%	0,0026
	f(mês)	-	7,74	< 0,001*		
	f(time)	-	6,69	< 0,005*		
Centro-Oeste	Intercepto	0,55	-	< 0,001*	0,42 / 46,3%	0,0021
	f(mês)	-	8,23	< 0,001*		
	f(time)	-	7,47	< 0,001*		
Brasil	Intercepto	0,70	-	< 0,001*	0,83 / 84,5%	0,0007
	f(mês)	-	8,50	< 0,001*		
	f(time)	-	5,53	< 0,001*		

R^2^: coeficiente de determinação ou de ajuste do modelo; DE: deviação explicada; CGV: validação cruzada generalizada; mês: mês do ano; time: número de meses ao longo do período de estudo.

* Significativo.

De maneira geral, as taxas de mortalidade apresentaram seus maiores valores em meados de cada ano, apontando para os meses de julho e agosto como os de maior risco, período de inverno no Hemisfério Sul, à exceção da região Norte, cujas capitais apresentaram os maiores picos entre março e maio. Por outro lado, as menores taxas se apresentaram no mês de fevereiro em todas as capitais das regiões geográficas e consequentemente no Brasil, exceto nas regiões Norte e Centro-Oeste.

Vale ressaltar que, com exceção do Norte do país, os meses de verão no Hemisfério Sul (dezembro a fevereiro) se caracterizaram por apresentar os menores valores das taxas de mortalidade por DCBV, entretanto, os meses de outono (abril a junho) e primavera (setembro a novembro) se comportam como períodos de transição (Figura 2).

## DISCUSSÃO

A maior frequência de óbitos em pessoas idosas confirma a idade como fator importante, associada frequentemente a múltiplos fatores de risco: aterosclerose vascular cerebral, hipertensão arterial (HTA), diabetes mellitus e síndrome metabólica, fumo, entre outros, cuja evolução, unida a fatores genéticos e imunológicos, contribuem com a deterioração da saúde^1^. Devido às falhas nas respostas de adaptação pelas mudanças internas dos sistemas regulatórios do organismo, os idosos também são mais suscetíveis na presença de ambientes com elevada carga de poluição e temperaturas extremas (mínimas e máximas)^9^.

No entanto, os adultos jovens também são alvo das DCBV^9,15^, com taxas de mortalidade não desprezíveis, como revelado neste estudo, apesar do declínio na tendência. Globalmente tem-se reportado incrementos na incidência em menores de 45 anos, com os maiores impactos em economias de baixa e média renda^15,16^. Estudos nacionais também caracterizaram a morbimortalidade cerebrovascular em jovens^14,17^, estando nas primeras causas específicas de morte de 5 a 29 e 30 a 69 anos de idade^14^. Em relação aos fatores de risco, estima-se que o aumento da incidência de causa cerebrovascular em adultos jovens é atribuída à tendência crescente dos múltiplos fatores de risco citados. Outros fatores etiológicos que favorecem a ocorrência de episódios cerebrovasculares nessas idades, também presentes no Brasil são: HIV, anemia falciforme, cardiopatia reumática, doença de Chagas, tuberculose, dissecção arterial e doença de moyamoya^14^.

Diversas pesquisas nacionais têm abordado amplamente a distribuição das DCBV associada ao sexo, à raça, ao estado civil, à escolaridade e anos de vida potencialmente perdidos (APVP)^4^. Lotufo et al.^18^, em um estudo sobre mortalidade cerebrovascular e raça, encontraram predomínio da raça negra e parda no Brasil, resultado que contrasta com os nossos, onde prodominou a branca, provavelmente pela restrição etária (30–69 anos) e o curto período de estudo selecionado por esse autor; no entanto, Dorlens et al.^2^reportaram as maiores taxas em idosos masculinos, solteiros e de baixa escolaridade entre 1996 e 2015, resultados similares aos nossos.

Três características decorrentes das análises permitem considerar o efeito protetor do hormônio estrogênio, o qual eventualmente contribui para a diminuição do risco de acidentes vasculares, aumentando a expectativa de vida na mulher: primeiramente, o predomínio das taxas de mortalidade nos homens de 30 a 59 anos, em comparação com o mesmo grupo em mulheres (Figuras 1B e 1F); em segundo lugar, as maiores reduções significativas da tendência de mortalidade em mulheres de 30 a 59 anos em relação aos homens da mesma faixa etária (Figuras 1B e 1F ); e por último o incremento das taxas de mortalidade em mulheres maiores de 60 anos em comparação com as menores de 60 anos (Figuras 1B e 1C). Ao estrogênio se atribuem efeitos positivos no sistema imune, estimulando a produção de anticorpos e regulando a linfopoiese; além disso, aumenta os níveis de colesterol HDL, reduz o LDL, e relaxa, suaviza e dilata os vasos sanguíneos. Outras propriedades são as antioxidantes, anti-inflamatórias e estabilizadoras das membranas celulares^15,19^.

No que se refere à tendência das taxas de mortalidade, nossos achados são concordantes com a literatura. Apesar do incremento no número absoluto de casos e da incidência em alguns estados e municípios do Brasil, de maneira geral se reporta um declínio nas taxas de mortalidade desde finais do século XX até 2017, sendo mais acentuado em regiões com maior desenvolvimento socioeconômico e avanços tecnológicos na medicina de alta complexidade^2^, comportamento também observado a escala global^1,16^. Nessa linha, foram as capitais das regiões Sul e Sudeste, de maior crescimento econômico, as que delinearam as maiores reduções dos APC e AAPC, ao contrário das regiões Norte e Nordeste, menos desenvolvidas economicamente.

Tomando como referência dados socioeconômicos e de recursos de saúde do Brasil de 2010, as regiões Norte e Nordeste eram as menores em produto interno bruto (PIB da RN = R$ 12,7; PIB da RND = R$ 9,6), em gastos per capita com ações e serviços públicos de saúde em geral (RN = R$ 572,88; RND = R$ 517,36), e em número de profissionais médicos (RN = 0,9/1.000 hab.; RND = 1,02/1.000 hab.), número de leitos hospitalares (RN = 194/1.000 hab.; RND = 2,29/1.000 hab.) e em número de aparelhos de neuroimagens para diagnóstico: tomógrafos (RN = 0,81 × 100.000 hab.; RND = 0,88 × 100.000 hab.) e ressonâncias magnéticas (RN = 0,05/100.000 hab.; RND = 0,02/100.000 hab.)^20^.

Por outro lado, a ampliação do acesso aos serviços de saúde e ao uso de medicamentos para a doença cerebrovascular aguda, doenças crônicas e transtornos lipídeos, bem como o maior controle sobre fatores de risco modificáveis, o incremento no número de unidades de cuidados intensivos, a disponibilidade de neuroimagem para diagnóstico e a implementação de programas de promoção e prevenção, são outros fatores que têm favorecido a redução das taxas de mortalidade^1,21,22^.

Nas regiões Norte e Nordeste a Pesquisa Nacional de Saúde (PNS)^23^ revelou fatores de risco para DCBV que se encontravam acima da média do país: proporção de adultos que nunca mediram sua pressão arterial (RN = 7%; RND = 4,2%), internações por HTA (RN = 14,6%; RND = 16%), internações por diabetes (RN = 14,2%; RND = 15,7%) e proporção de pessoas com diagnóstico prévio de DCBV (RN = 29,7%; RND = 26,4%). Outros fatores de risco prejudiciais com indicadores abaixo da média nacional foram: proporção de adultos hipertensos que tiveram acesso no mínimo a um medicamento no Programa Farmácia Popular (RN = 35%; RND = 28,7%), idade média de início do consumo de álcool (RN = 18,5 anos; RND = 18,3 anos) e proporção de pessoas com prática de atividade física recomendada (RN = 22,2 %; RND = 22,3%).

Lee et al.^24^ encontraram achados similares na Coreia do Sul (2011–2015), descrevendo aumento das taxas de mortalidade cerebrovascular em regiões de menor desenvolvimento socioeconômico, assim como associação com sobrepeso, consumo de álcool, número de leitos hospitalares e número de aparelhos de neuroimagem disponíveis para a população. Yanez et al.^25^na Colômbia também referiram incrementos nas regiões mais pobres, com maiores dificuldades de acesso aos serviços de saúde pré-hospitalar e hospitalar, além da correlação com HTA, obesidade e tabagismo. Porém no município de Iwate (Japão) Omama et al.^22^ reportaram queda na tendência das taxas de incidência por doenças cerebrovasculares entre 2008 e 2017 em maiores e menores de 55 anos de ambos os sexos.

No concernente ao comportamento sazonal, a elevação das taxas em meados de cada ano nos remete, desde o ponto de vista climático, a que no período de inverno, exceto nas capitais da RN, o risco de mortalidade cerebrovascular aumenta, especificamente durante os meses de julho e agosto. Nessa época do ano se registram as temperaturas regionais mais baixas do país^26^. No entanto, nas capitais da RN, excluindo sua parte meridional, o padrão do comportamento das taxas difere, provavelmente porque durante esse período a região não apresenta temperaturas mínimas importantes, as que são bem mais altas (18ºC a 23ºC) do que no resto do país^26^.

No período de inverno, múltiplos são os mecanismos fisiopatológicos ligados à ocorrência de óbitos cardiovasculares: aumento dos níveis de catecolaminas, vasoconstrição, taquicardia e aumento da pressão arterial, hemoconcentração devido à poliúria relacionada ao frio, aumento da viscosidade sanguínea por concentrações de fatores de coagulação, plaquetas, colesterol, fibrinogênio e eritrócitos^9^.

Cabe destacar que no verão, embora as taxas apresentem seus valores mais baixos, o risco está presente. O incremento da mortalidade em condições de clima quente se associa às temperaturas extremas e ondas de calor, com consequências negativas mais acentuadas em pessoas idosas^9^. As capitais da RN se afastam do comportamento do resto das capitais do país, acompanhando a mudança do padrão climático, uma vez que setembro e outubro, meses mais quentes do ano nessa região^27^, coincidem com as menores taxas de óbito cerebrovascular, ao contrário das outras regiões, cujo declínio ocorre de dezembro a fevereiro. No entanto, a localização geográfica das capitais entre as zonas equatorial, tropical e subtropical sugere que a população, constantemente exposta às mudanças ambientais, se adapta melhor às temperaturas quentes do que às frias, independente de que outros fatores não explorados nesta pesquisa, podem estar determinando esse comportamento.

Em concordância com nossos resultados, tanto Keatinge et al.^27^ em várias regiões europeias quanto Su et al.^28^ em 17 cidades chinesas reportaram maior número anual de óbitos e maior risco relativo de mortalidade cardiovasculares relacionadas ao frio, ainda estabelecendo diferenças regionais e apontando para pequena proporção de óbitos não relacionados à variabilidade sazonal.

Silveira et al.^29^, no Brasil, concluíram que, tanto as baixas quanto as altas temperaturas acrescentam o risco de mortalidade cardiovascular na maioria das capitais brasileiras, com variações de acordo à localização geográfica. Em geral, foi observada uma relação exposição-resposta em forma de U, com consequências mais acentuadas em condições de baixas temperaturas e locais com maior amplitude térmica.

Acerca de potenciais limitações desta pesquisa, vale apontar que o uso de dados secundários, sujeitos a diferentes níveis de qualidade relacionados à subnotificação e causas mal definidas, pode ter influenciado parcialmente nos resultados. Neste particular, no que tange à abrangência, cobertura e veracidade, estudos confirmam avanços importantes na qualidade dos dados de mortalidade no Brasil, com destaque para as áreas de maior desenvolvimento socioeconômico^2^. Acredita-se que os dados gerados pelo Sistema de Informação de Mortalidade (SIM) são capazes de reproduzir a dinâmica espaço-temporal sobre a situação de mortalidade, gerando subsídios e prioridades para políticas e ações à promoção de saúde^14^. Outra limitação a ser considerada é que não foram especificados os tipos de vasculares cerebrais, mesmo havendo diferentes mecanismos fisiopatológicos entre eles.

Também constitui uma limitação a abordagem dos dados agrupados por regiões, o que provavelmente não permite captar com precisão comportamentos da mortalidade a nível local. No entanto, considera-se que o estudo estima as mudanças significativas da tendência e sazonalidade, permitindo sintetizar características de interesse para a saúde pública relacionada à mortalidade cerebrovascular, em áreas de alta concentração populacional e sujeitas à influência de fatores comuns, tanto ambientais como sociais e econômicos.

Conclui-se que os achados permitiram caracterizar o perfil epidemiológico e sociodemográfico nas capitais brasileiras e o DF quanto à mortalidade cerebrovascular entre 2000–2019, apontando principalmente as pessoas idosas de ambos os sexos, solteiras e com baixa escolaridade como as mais atingidas e, portanto, com maior grau de vulnerabilidade social. A depender da região, predominou a raça branca (Sul, Sudeste) e preta (Norte, Nordeste e Centro-Oeste). Na avaliação da tendência das taxas de mortalidade, embora venha reduzindo ao longo do período, seu comportamento ainda preocupa, devido ao caráter multifatorial da sua origem e evolução. A análise da sazonalidade mostrou o inverno como o período de maior risco de mortalidade no território nacional. As diferenças regionais permitem definir prioridades e subsidiar os tomadores de decisões, com ações de médio e longo prazo, em relação ao planejamento e implementação de políticas e programas de redução, promoção e prevenção da mortalidade cerebrovascular.

Contudo, o estudo ressalta a necessidade de aprofundar e ampliar as pesquisas considerando outros fatores de risco que não fizeram parte do escopo deste estudo.
